# Supplementary material for: Role and mechanism of NCAPD3 in promoting malignant behaviors in gastric cancer
Source: Front Pharmacol. 2024 Apr 22;15:1341039. doi: 10.3389/fphar.2024.1341039 (PMC11070777; doi:10.3389/fphar.2024.1341039)
Supplement: Supplementary file 11 [file DataSheet2.ZIP › GSEA/Transcipt factor/my_analysis.Gsea.1599461814734/ADA2_TARGET_GENES.html]

Details for gene set ADA2\_TARGET\_GENES[GSEA]

|  || Dataset | filtered\_dataset.sample\_info.cls#WT\_versus\_NCAPD3\_MUT |
| Phenotype | sample\_info.cls#WT\_versus\_NCAPD3\_MUT |
| Upregulated in class | WT |
| GeneSet | ADA2\_TARGET\_GENES |
| Enrichment Score (ES) | 0.30961066 |
| Normalized Enrichment Score (NES) | 1.9792002 |
| Nominal p-value | 0.0 |
| FDR q-value | 0.18057516 |
| FWER p-Value | 0.687 |
Table: GSEA Results Summary

  

Fig 1: Enrichment plot: ADA2\_TARGET\_GENES      
 Profile of the Running ES Score & Positions of GeneSet Members on the Rank Ordered List

  

| SYMBOL | TITLE | RANK IN GENE LIST | RANK METRIC SCORE | RUNNING ES | CORE ENRICHMENT || 1 | 51111 | KMT5B | 2 | 1.217 | 0.0265 | Yes |
| 2 | 3954 | LETM1 | 47 | 0.924 | 0.0146 | Yes |
| 3 | 388536 | ZNF790 | 52 | 0.913 | 0.0326 | Yes |
| 4 | 54149 | C21orf91 | 62 | 0.864 | 0.0457 | Yes |
| 5 | 117145 | THEM4 | 65 | 0.860 | 0.0640 | Yes |
| 6 | 10207 | INADL | 73 | 0.851 | 0.0783 | Yes |
| 7 | 157680 | VPS13B | 78 | 0.838 | 0.0945 | Yes |
| 8 | 50861 | STMN3 | 126 | 0.777 | 0.0771 | Yes |
| 9 | 55687 | TRMU | 128 | 0.774 | 0.0941 | Yes |
| 10 | 55900 | ZNF302 | 145 | 0.740 | 0.0991 | Yes |
| 11 | 51434 | ANAPC7 | 166 | 0.710 | 0.1004 | Yes |
| 12 | 54971 | BANP | 172 | 0.707 | 0.1129 | Yes |
| 13 | 23450 | SF3B3 | 178 | 0.697 | 0.1252 | Yes |
| 14 | 51018 | RRP15 | 192 | 0.685 | 0.1312 | Yes |
| 15 | 6191 | RPS4X | 196 | 0.682 | 0.1446 | Yes |
| 16 | 84142 | FAM175A | 211 | 0.667 | 0.1494 | Yes |
| 17 | 146050 | ZSCAN29 | 226 | 0.654 | 0.1539 | Yes |
| 18 | 5701 | PSMC2 | 244 | 0.636 | 0.1557 | Yes |
| 19 | 152485 | ZNF827 | 268 | 0.618 | 0.1527 | Yes |
| 20 | 403313 | PLPP6 | 274 | 0.615 | 0.1631 | Yes |
| 21 | 80124 | VCPIP1 | 282 | 0.607 | 0.1718 | Yes |
| 22 | 25972 | UNC50 | 297 | 0.593 | 0.1749 | Yes |
| 23 | 647087 | C7orf73 | 308 | 0.588 | 0.1809 | Yes |
| 24 | 26259 | FBXW8 | 318 | 0.583 | 0.1875 | Yes |
| 25 | 94107 | TMEM203 | 333 | 0.574 | 0.1902 | Yes |
| 26 | 51491 | NOP16 | 334 | 0.573 | 0.2034 | Yes |
| 27 | 4297 | KMT2A | 337 | 0.573 | 0.2150 | Yes |
| 28 | 9617 | MTRF1 | 349 | 0.568 | 0.2198 | Yes |
| 29 | 55869 | HDAC8 | 352 | 0.566 | 0.2313 | Yes |
| 30 | 9315 | NREP | 364 | 0.557 | 0.2359 | Yes |
| 31 | 57109 | REXO4 | 370 | 0.552 | 0.2448 | Yes |
| 32 | 9786 | KIAA0586 | 379 | 0.547 | 0.2514 | Yes |
| 33 | 10426 | TUBGCP3 | 402 | 0.535 | 0.2472 | Yes |
| 34 | 11019 | LIAS | 409 | 0.533 | 0.2549 | Yes |
| 35 | 54665 | RSBN1 | 429 | 0.516 | 0.2525 | Yes |
| 36 | 8607 | RUVBL1 | 451 | 0.506 | 0.2483 | Yes |
| 37 | 9690 | UBE3C | 470 | 0.493 | 0.2462 | Yes |
| 38 | 6742 | SSBP1 | 501 | 0.472 | 0.2345 | Yes |
| 39 | 1819 | DRG2 | 550 | 0.447 | 0.2087 | Yes |
| 40 | 27229 | TUBGCP4 | 556 | 0.442 | 0.2151 | Yes |
| 41 | 6632 | SNRPD1 | 563 | 0.440 | 0.2207 | Yes |
| 42 | 27349 | MCAT | 574 | 0.436 | 0.2232 | Yes |
| 43 | 55245 | UQCC1 | 580 | 0.434 | 0.2295 | Yes |
| 44 | 93134 | ZNF561 | 590 | 0.431 | 0.2326 | Yes |
| 45 | 4548 | MTR | 592 | 0.428 | 0.2417 | Yes |
| 46 | 55181 | SMG8 | 602 | 0.422 | 0.2446 | Yes |
| 47 | 6208 | RPS14 | 604 | 0.421 | 0.2536 | Yes |
| 48 | 7290 | HIRA | 612 | 0.417 | 0.2579 | Yes |
| 49 | 84948 | TIGD5 | 619 | 0.413 | 0.2629 | Yes |
| 50 | 54512 | EXOSC4 | 623 | 0.411 | 0.2700 | Yes |
| 51 | 5147 | PDE6D | 624 | 0.410 | 0.2795 | Yes |
| 52 | 643836 | ZFP62 | 639 | 0.405 | 0.2783 | Yes |
| 53 | 79807 | GSTCD | 644 | 0.402 | 0.2845 | Yes |
| 54 | 78996 | C7orf49 | 661 | 0.396 | 0.2816 | Yes |
| 55 | 5134 | PDCD2 | 678 | 0.388 | 0.2785 | Yes |
| 56 | 6427 | SRSF2 | 679 | 0.388 | 0.2874 | Yes |
| 57 | 9831 | ZNF623 | 691 | 0.383 | 0.2880 | Yes |
| 58 | 89910 | UBE3B | 693 | 0.381 | 0.2960 | Yes |
| 59 | 55750 | AGK | 697 | 0.380 | 0.3024 | Yes |
| 60 | 79670 | ZCCHC6 | 700 | 0.378 | 0.3096 | Yes |
| 61 | 84172 | POLR1B | 718 | 0.365 | 0.3052 | No |
| 62 | 134430 | WDR36 | 733 | 0.359 | 0.3030 | No |
| 63 | 9223 | MAGI1 | 737 | 0.357 | 0.3089 | No |
| 64 | 3146 | HMGB1 | 831 | 0.295 | 0.2459 | No |
| 65 | 2872 | MKNK2 | 860 | -0.269 | 0.2310 | No |
| 66 | 10589 | DRAP1 | 882 | -0.300 | 0.2222 | No |
| 67 | 7453 | WARS | 900 | -0.325 | 0.2169 | No |
| 68 | 23608 | MKRN1 | 927 | -0.352 | 0.2054 | No |
| 69 | 8881 | CDC16 | 963 | -0.383 | 0.1880 | No |
| 70 | 100534599 | ISY1-RAB43 | 1082 | -0.462 | 0.1100 | No |
| 71 | 54541 | DDIT4 | 1133 | -0.502 | 0.0840 | No |
| 72 | 604 | BCL6 | 1136 | -0.504 | 0.0941 | No |
| 73 | 22931 | RAB18 | 1231 | -0.608 | 0.0375 | No |
| 74 | 10106 | CTDSP2 | 1239 | -0.618 | 0.0464 | No |
| 75 | 26137 | ZBTB20 | 1244 | -0.622 | 0.0577 | No |
| 76 | 5187 | PER1 | 1254 | -0.641 | 0.0657 | No |
| 77 | 2355 | FOSL2 | 1262 | -0.648 | 0.0753 | No |
| 78 | 8916 | HERC3 | 1269 | -0.660 | 0.0860 | No |
| 79 | 26136 | TES | 1361 | -0.833 | 0.0368 | No |
Table: GSEA details [plain text format]

  

Fig 2: ADA2\_TARGET\_GENES      
 Blue-Pink O' Gram in the Space of the Analyzed GeneSet

  

Fig 3: ADA2\_TARGET\_GENES: Random ES distribution      
 Gene set null distribution of ES for **ADA2\_TARGET\_GENES**
